# Acousto-Electronic Sensor Based on Langmuir-Blodgett Films of Tetra-Tert-Butylphthalocyaninate Zinc for Chemical Vapor Detection

**DOI:** 10.3390/s25227069

**Published:** 2025-11-19

**Authors:** Ilya Gorbachev, Andrey Smirnov, Vladimir Kolesov, Alexey Yagodin, Alexander Martynov, Yulia Gorbunova, Iren Kuznetsova

**Affiliations:** 1 Kotelnikov Institute of Radio Engineering and Electronics of RAS, Moscow 125009, Russia; sareth221@yandex.ru (I.G.); andre-smirnov-v@yandex.ru (A.S.); kvv@cplire.ru (V.K.); 2 Frumkin Institute of Physical Chemistry and Electrochemistry Russian Academy of Sciences, 31-4, Leninsky Prospect, Moscow 119071, Russia; al.yagodin@mail.ru (A.Y.); martynov@phyche.ac.ru (A.M.); yulia@igic.ras.ru (Y.G.); 3 Kurnakov Institute of General and Inorganic Chemistry Russain Academy of Science, 31-4, Leninsky Prospect, Moscow 119991, Russia

**Keywords:** acousto-electronic sensor, surface acoustic waves, Langmuir-Blodgett films, tetra-tert-butylphthalocyaninatozinc, chloroform, selective detection, film morphology

## Abstract

In this work, the sensor properties of multilayered Langmuir-Blodgett (LB) films of tetra-tert-butylphthalocyaninate zinc (tBuZnPc) were studied using an acoustoelectronic method. The morphology and optical properties of the fabricated films were characterized by atomic force microscopy and ultraviolet-visible spectroscopy, respectively. The LB films were deposited on surface acoustic wave (SAW) delay lines, and their gas-sensing properties were investigated. The films demonstrated high selectivity towards chloroform vapor compared to acetone, methanol, ethanol, and isopropanol. The highest selectivity was observed for the five-layer film, which can be attributed to the specific interaction of chloroform molecules with the hydrophobic cavities formed by the tert-butyl groups. Increasing the film thickness to 41 layers enhanced the absolute response to chloroform to 370 ppm; however, the selectivity decreased due to increased nonspecific adsorption. The results demonstrate the potential of using tBuZnPc LB films as sensitive coatings for the selective detection of chloroform in environmental and industrial monitoring applications.

## 1. Introduction

Rapid advancements in the chemical industry have led to a substantial increase in potential sources of environmental pollution. The necessity to comply with strict environmental regulations has driven intensive research efforts aimed at developing new types of sensors capable of detecting a wide range of environmental pollutants. Chloroform is one widely used chemical substance in manufacturing processes. It is applied in the production of synthetic organic compounds, pharmaceuticals, and polymeric materials. It is also frequently a component of multi-component mixtures alongside ethanol, acetone, cyclohexane, and other substances [1]. Current devices for detecting the presence and concentration of chloroform are based on various physical principles, such as chemoresistive, potentiometric, optical, and acoustoelectronic sensing, along with their modifications. Despite the wide variety of existing devices, they often lack sufficient selectivity for the target compound. Therefore, a significant area of research focuses on the development of specialized sensor coatings. In the article [2], a sensitive element based on TiO_2_ nanotubes was developed for a low-temperature chloroform sensor. The resulting sensor demonstrated sensitivity to chloroform vapor in the concentration range of 1000 to 20,000 ppm. However, a significant drawback of the proposed design was its dependence on the ambient humidity level. By authors [3] a composite material based on transition metal oxides and multi-walled carbon nanotubes deposited on a carbon electrode was used as a sensor layer. The developed sensor exhibited sensitivity to chloroform vapors in the concentration range from 3.5 nM to 35 mM. The authors [4] investigated the sensory properties of poly(3-decylthiophene-2,5-diyl) polymer films prepared using the drop-casting method. The results demonstrate the potential of these films for use in electrochemical sensors for chloroform vapor. In work [5], an optical sensor for the trace detection of chloroform in aqueous and non-aqueous solutions was developed based on the Fujiwara reaction. The sensor’s sensitivity reached 0.830 ppm in non-aqueous solutions and 500 ppm in aqueous solutions. For its fabrication, 2,2′-dipyridyl and tetrabutylammonium hydroxide (TBAH) were encapsulated in an ethylcellulose film. The advantages of this approach include its suitability for field testing, as the sensor exhibits a color change in the visible range. In the article [6], a film was fabricated by spin-coating from a solution of the synthesized material, 1,2,4,5-tetraphenyl-1,4-dihydropyrrolo[3,2-b]pyrrole. Investigation of the film’s response to various volatile organic compound vapors revealed a selective fluorescent response to chloroform. It was demonstrated that this film can be used for the quantitative analysis of chloroform, achieving a detection limit of 100 ppm. Although such sensor coatings exhibit high sensitivity and selectivity, their widespread application is hindered by the complex fabrication process. Works [7,8] A simpler method for creating a sensor coating selective to chloroform vapor is described, utilizing the Langmuir-Blodgett (LB) technique. Multilayer films of arachidic acid were deposited onto a surface acoustic wave (SAW) acoustoelectronic resonator to form the sensing layer. The resulting device enabled the detection of chloroform vapor over a wide concentration range. A key advantage of the developed sensor is the versatility of the fabrication technologies employed.

The Langmuir-Blodgett (LB) technique is a versatile method for fabricating highly structured organic coatings with precisely controlled thickness, down to a single molecular monolayer. This technology enables the formation of coatings from diverse materials, including phospholipid molecules with incorporated enzymes [9,10], polymer molecules [11], as well as inorganic nano- and microparticles [12]. This opens up opportunities for creating various types of coatings that are sensitive to changes in the acidity of the environment [13] and lighting conditions [14]. Furthermore, this technology enables the fabrication of thin, highly sensitive coatings that are compatible with high-frequency acoustoelectronic devices. In a typical sensor configuration, a sensitive coating is applied to the surface of a surface acoustic wave (SAW) delay line, specifically in the propagation path between the interdigital transducers (IDTs). A surface acoustic wave generated by the transmitting IDT travels along the substrate surface to the receiving IDT, interacting with the coating at their interface. The acoustic wave parameters—specifically, the signal’s amplitude and phase, measured by a vector network analyzer—are highly dependent on the properties of this coating. Changes in the atmospheric composition cause chemical or physical adsorption of analyte molecules onto the coating, altering its properties such as elastic modulus, density, viscosity, and electrical conductivity. These changes, in turn, induce corresponding shifts in the amplitude and phase of the acoustic signal. A key feature of such devices is that the sensitivity to changes in the surface state increases with the operating frequency of the delay line. However, the application of a sensitive coating to the substrate surface adversely affects the acoustic wave propagation. If an excessively thick film is formed, the acoustic signal can be completely attenuated before reaching the receiving transducer. Therefore, in the design of high-frequency SAW delay line-based sensor elements, precise control over the coating thickness is essential to balance the acoustic losses introduced by the coating with the overall sensitivity of the sensor. Given these requirements, the combination of Langmuir-Blodgett technology with high-frequency surface acoustic wave (SAW) delay lines presents a promising approach for developing highly sensitive and selective sensor elements for environmental analysis.

This approach has demonstrated its efficacy in prior research. For instance, a study [15], reported the creation of a Langmuir-Blodgett film with immobilized alcohol oxidase enzyme on the surface of an acoustoelectronic delay line for a selective ethanol vapor sensor. The resulting device could detect ethanol vapor in dry air at volume concentrations from 0% to 100%. A similar methodology was applied to fabricate a glucose sensor for aqueous solutions. In that work [16], a film incorporating glucose oxidase enzyme was formed on the surface of an acoustic delay line. The sensor exhibited sensitivity to glucose concentrations ranging from 0 to 1 mg/mL

Phthalocyanines and their derivatives are promising materials for sensor coatings. Their molecules exhibit high chemical activity and effectively interact with toxic gases and vapors of various volatile organic compounds. For instance, in study [17], sensor coatings based on zinc and copper phthalocyanines were deposited onto the surface of a quartz crystal microbalance (QCM) using physical vapor deposition. The resulting films demonstrated sensitivity to vapors of ethanol, acetone, formaldehyde, and toluene, with the highest response observed for ethanol. Similarly, in work [18], Langmuir-Blodgett films of iron and copper phthalocyanines were deposited on a surface acoustic wave (SAW) resonator, creating a nitrogen dioxide sensor with a sensitivity of up to 40 ppb.

It should be noted that alternative approaches to creating acoustoelectronic chloroform sensors are not well detailed in the literature. For example, a study [19] reported an acousto-optic chloroform vapor sensor based on a resonant chamber and a microphone array for audio signal detection. This sensor operated on the principle of photoacoustic spectroscopy. A distributed feedback (DFB) laser with a wavelength of 1683 nm was used to excite the chloroform molecules. The laser radiation induced localized heating and the decomposition of the chloroform molecules, which generated sound waves at a frequency of 6.39 kHz. The sound waves were captured by the microphone array and the signal was demodulated, enabling a high sensitivity of 0.28 ppm. The primary drawback of this method is its significant technological complexity. In a different study [20], a SAW sensor based on a porous gold film with an immobilized acetylcholinesterase enzyme was developed. The sensitivity of this sensor reached 9–12 Hz/ppm. Its selectivity was achieved through the enzyme’s specific binding with chloroform. Similarly to the previous approach, a major limitation is the complex fabrication process, which stems from the need to maintain enzyme activity during both immobilization and the sensor’s operational lifetime. Comparative characteristics of the described sensors are provided in Table 1.

When creating phthalocyanine films, one of the most important aspects is controlling their supramolecular organization within the film. This ensures that target molecules have access to the appropriate part of the phthalocyanine molecule [21,22,23]. A highly effective strategy for tuning the properties of phthalocyanines, including their supramolecular organization, is to modify their molecular structure by introducing functional substituents [24], including their supramolecular organization, is the modification of their molecular structure through the introduction of functional substituents. The incorporation of suitable substituents enhances the solubility of phthalocyanine molecules in solvents of different polarity [25,26,27]. This facilitates the formation of stable Langmuir monolayers due to changes in intermolecular interactions at the air–water interface. Phthalocyanines bearing tert-butyl substituents are particularly promising for use in Langmuir–Blodgett (LB) technology. The bulky *t*-Bu groups improve solubility in nonpolar organic solvents (e.g., chloroform) and hinder tight π–π stacking. This effect is associated with their increased solubility in chloroform [28]. Moreover, in contrast to unsubstituted phthalocyanines, the presence of such substituent groups reduces spontaneous stacking, thereby improving the ability to control their supramolecular organization within Langmuir monolayers [29,30].

The formation of thin phthalocyanine films using the Langmuir-Blodgett technique on quartz-based acousto-electronic devices is relatively straightforward. However, fewer studies have focused on developing acousto-electronic sensors based on plate acoustic waves, particularly for chloroform vapor detection. This can be partly attributed to the need to refine the transfer technology of Langmuir-Blodgett films onto the surfaces of acoustic delay lines made from piezoelectric materials such as LiNbO_3_ or LiTaO_3_.

Given the aforementioned considerations, the task of creating a selective sensor element capable of detecting chloroform vapor in a gas-air mixture remains relevant. The objective of this study was to investigate the sensory properties of a tetra-tert-butylphthalocyanine zinc film using an acousto-electronic delay line based on surface acoustic waves when exposed to various volatile chemical vapors.

## 2. Experimental Part

### 2.1. Formation of Langmuir Monolayers and Langmuir-Blodgett Films of Tetra-Tert-Butylphthalocyaninate Zinc

All experiments involving the study of surface properties, formation, and transfer of tetra-tert-butylphthalocyanine zinc Langmuir monolayers onto solid substrates were conducted using a Nima KSV LB Trough KN2001 apparatus (Nima KSV, Espoo, Finland). Before the start of the monolayer formation process, the surface of the Langmuir trough was sequentially cleaned with ethyl alcohol, chloroform, and deionized water. Subsequently, the trough was filled with deionized water with a resistivity of 18 MΩ·cm. The water temperature was maintained at 24 °C using a Termex A160 thermostat (Termex, Tosno, Russia). The pH of the water was 6.5.

The Langmuir monolayers were formed from a solution of tetra-tert-butylphthalocyanine zinc (tBuZnPc) in chloroform at a concentration of 7 × 10^−7^ M/L. For this purpose, an aliquot of the tBuZnPc solution with a volume of 75 µL was applied to the surface of the aqueous subphase. After a 20-min equilibration period, the monolayer was compressed using mobile barriers at a constant area reduction rate of 7 cm^2^/min. The compression isotherm, which reflects the change in surface pressure as a function of area, was recorded automatically.

**Figure 1:**
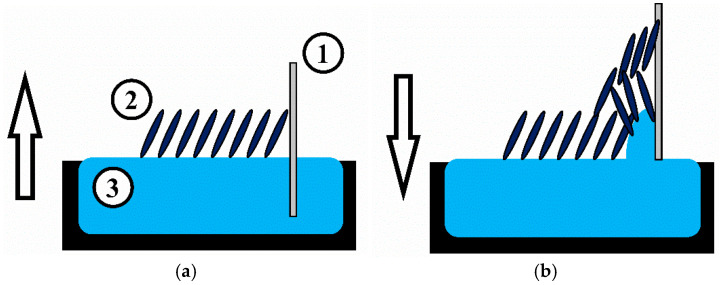
Schematic representation of the Langmuir-Blodgett film formation process (**a**) and transferring of the first layer on the solid substrate (**b**), where (1) is the LiNbO_3_ acoustic delay line, (2) is the tetra-tert-butylphthalocyaninate zinc monolayer, and (3) is the aqueous subphase. The arrows indicate the direction of substrate movement during the transfer process.

It is known that a phase transition in a monolayer is accompanied by a change in its compressibility [31]. The compressibility of the monolayer was calculated using the following expression (1):

(1) $$C_{s}=-A_{0}\frac{d\pi }{dA}$$

where *Cs* is the compressibility of the monolayer, *A*_0_ is the specific area per molecule in the non-tilted condensed phase, and *dπ/dA* is the numerical value of the derivative of surface pressure with respect to the area per molecule. Figure 1 also shows the dependence of the tBuZnPc monolayer compressibility on the specific area per molecule.

Monolayers were transferred onto solid substrates to form sensor films using the Langmuir-Blodgett technique. A schematic illustration of the tBuZnPc Langmuir-Blodgett film formation and transfer process is shown in Figure 1.

The substrates used were acoustic delay lines fabricated on piezoelectric lithium niobate (LiNbO_3_) plates and double-sided polished LiNbO_3_ plates without interdigital transducers (IDTs). The monolayer transfer was performed using the following procedure. The acoustic delay line was immersed into the water until the distance between the upper pair of IDTs and the water surface reached 1 mm. A Langmuir monolayer of tBuZnPc was then formed on the aqueous subphase. When the surface pressure reached 19.5 mN/m, the substrate was withdrawn from the water at a constant speed of 1 mm/min until the distance between the lower pair of IDTs and the water surface was 1 mm.

The monolayer was dried in this position for 5 min. Afterward, the substrate was submerged again until the distance between the upper pair of IDTs and the water surface reached 1 mm. Using this method, sensor films comprising 5, 11, 21, and 41 tBuZnPc monolayers were formed. The resulting films were then dried under a bell jar at 24 °C for 4 h. To study the morphology by atomic force microscopy and the absorption spectra, tBuZnPc films were also formed on double-sided polished LiNbO_3_ plates.

### 2.2. Study of Film Morphology Using Atomic Force Microscopy

Langmuir monolayers of tBuZnPc were transferred onto solid substrates made of polished YX-128^0^ LiNbO_3_ plate (Fomos materials, Moscow, Russia) using the Langmuir-Blodgett method to study their surface morphology. Films containing 5, 11, 21, and 41 Langmuir monolayers of ZnPc were formed. The number of film layers was increased in a geometric progression with a factor of 2. This selection was made to cover a wide range of thicknesses with a relatively limited number of samples. The use of a geometric progression helps identify qualitative changes in the sensor’s response and determine potential transition regions between different adsorption mechanisms of the studied compounds. This approach allows for the investigation of the film thickness influence on its optical and acoustic properties over a wide range. The use of an odd number of layers is related to the technological specifics of depositing the first layer from the subphase, which may have incomplete coverage and a different density compared to subsequent layers. The morphology of the films was studied using atomic force microscopy on an NT-MDT Ntegra Prima system (NT-MDT, Zelenograd, Russia). The measurements were performed in the hybrid mode with a scan rate of 0.7 Hz per line, using an NSG01_DLC cantilever with a resonant frequency of 170 kHz and a tip curvature radius of less than 3 nm. Mathematical processing and analysis of the obtained images were performed using Gwyddion 2.65 software. The surface roughness parameters of the films were calculated from this processing. The roughness was calculated using the following expression (2):

(2) $$S_{a}=\frac{1}{N}\sum_{j=1}^{N}\left|r_{j}\right|$$

where *Sa* is the arithmetic mean roughness, calculated as the average absolute deviation of the profile from the mean line. It is defined as the sum of the absolute values of the profile deviations (*rj*) measured at a set of discrete points (*N*) along the evaluation length, divided by the number of points.

The average surface area of the film (*P*) was calculated automatically using Gwyddion 2.65 software. This calculation involved dividing the surface image into right triangles and computing their individual areas. The total surface area was then determined as the sum of the areas of all the triangles.

### 2.3. Fabrication of Acoustic Delay Line on Surface Acoustic Waves

**Figure 2:**
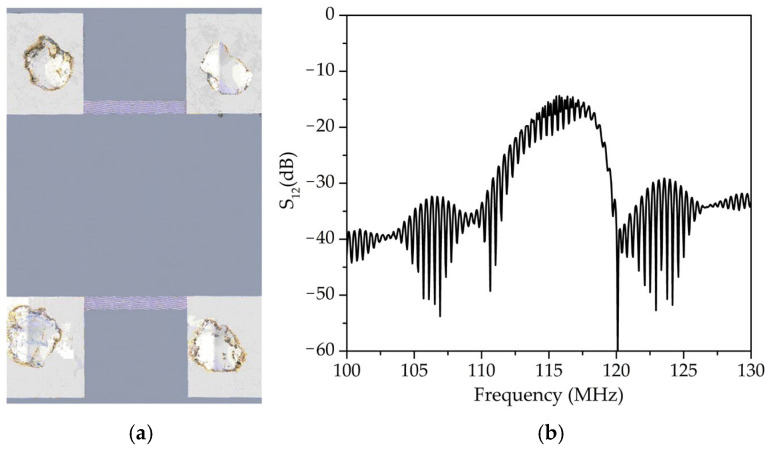
Photograph of the used acoustic delay line (**a**) and its amplitude-frequency characteristic (**b**).

The acousto-electronic delay line was fabricated using projection photolithography and DC magnetron sputtering. The substrates used were 128° YX-LiNbO_3_ piezoelectric plates measuring 25 × 15 mm with a thickness of 3 mm. The electrode structures were formed on the substrate surface using a VSE-PVD-desk-pro system (Novosibirsk, Russia), with aluminum (aluminum target grade A995, purity 99.995% wt., LLC “GIROMET” TU 28.29.70-013-56631876-2017) used as the material. The coating thickness was monitored using a quartz sensor inside the vacuum chamber and a Model 130 profilometer (Proton Plant, Tomsk, Russia). The roughness and thickness parameters of the coatings were additionally verified with an Olympus LEXT laser confocal microscope. Substrate preparation for coating deposition consisted of two stages: the first stage involved chemical cleaning with ethanol, acetone, and Piranha solution; the second stage involved argon ion treatment using a multi-cell ion source with closed electron drift and an APEL-IS-3500 A power supply with Uout = 0.35–3.5 kV, Iout = 6–600 mA (Applied Electronics LLC, Tomsk, Russia). The chamber pressure was maintained at 5.4 × 10^−4^ Torr. A photoresist mask was created on the surface of the piezoelectric material using projection photolithography with a Smart Print machine (Microlight 3D, Grenoble, France). The method eliminates the need for a physical photomask. Instead, a pattern is formed using a monochromatic display matrix and an optical system with interchangeable objectives to vary the minimum feature size of the fabricated structures. The photoresist layer was formed using a Sawatec SM-180-BT (Sawatec AG, Sax, Switzerland) spin coater. A positive photoresist (FP-3515) was used. The photoresist was baked on a Heidolph hotplate. A virtual photomask was created using the free software Layout. The exposed areas of the photoresist were removed using a metal-free developer (P-236A-MF). The quality of the fabricated electrode structures was verified using an Olympus LEXT laser confocal microscope. Figure 2 shows an image of the fabricated acousto-electronic delay line and its amplitude-frequency characteristic. The transducer wavelength (λ) was 34 μm, and the distance between the transducers (L) was 9 mm. The center frequency was 116.7 MHz.

### 2.4. Study of Absorption Spectrum of Tetra-Tert-Butylphthalocyaninate Zinc Films

The absorption spectra of Langmuir-Blodgett tBuZnPc films were studied using a dual-beam UV-Vis spectrophotometer PG Instruments T70 (PG Instruments, Wibtoft, UK). The spectral measurement range was from 190 to 1100 nm with a bandwidth of 0.5 nm. 

### 2.5. Study of the Sensory Properties of the Formed Films on a Gas Sensitivity Study Setup

**Figure 3:**
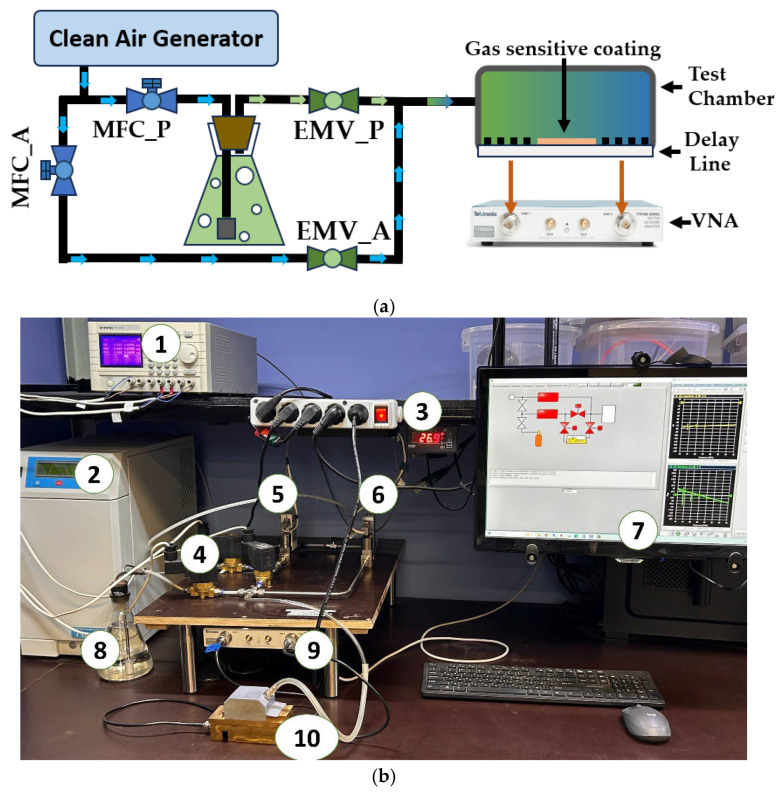
Schematic representation of the automated test bench for studying gas-sensitive properties (**a**) and a photo (**b**), where 1 is a power supply, 2 is a clear air generator, 3 is a temperature sensor, 4, 5, 6 is a mass flow control (MFC), 7 is a work station with control software, 8 is a studied liquid, 9 is a vector network analyzer, and 10 is a hermetic chamber with acoustic delay line.

The gas sensitivity of the fabricated tBuZnPc films was measured three times for each VOC using an automated test bench. The measurement error for the response magnitude was approximately 5–7%. A schematic representation of the setup is shown in Figure 3.

The test stand operation begins with a clean air generator (CAG), which produces a flow of pure, dry, catalytically purified air. This flow is then split into two separate streams. The flow rate of each stream is regulated using Bronkhorst Prestige series thermal mass flow controllers (MFCs). The air stream passing through the MFC_P controller is directed to bubble through the test liquid, resulting in the formation of saturated vapor of the test substance. This vapor is then mixed with the dry air stream passing through the MFC_A controller. The resulting gas-vapor mixture enters the test chamber, which contains the acousto-electronic delay line with the deposited test film on its surface. The desired concentration of the test substance is achieved by varying the ratio of the dry air flow to the test substance vapor flow (ethanol, acetone, etc.). EMV_P and EMV_A are electromagnetic shut-off valves.

Data on changes in the insertion loss magnitude and the acoustic wave phase were collected and analyzed using a Tektronix TTR-506A (Tektronix, Beaverton, OR, USA) vector network analyzer. The analyzer was connected to a personal computer via a USB interface, enabling real-time processing and analysis of the collected data. This system provides a comprehensive approach for studying the interaction between the sensor film and the saturated vapor of the test liquid.

The measurement chamber and fixture components were fabricated using 3D printing technology (Phrozen Sonic Mini 8K, Hsinchu City, Taiwan). Anycubic Basic Water-washable photopolymer resin (Anycubic, Shenzhen, China) was used for this purpose. To remove uncured photopolymer residue from the chamber surface, the chamber was immersed in an isopropyl alcohol solution and ultrasonically treated for 30 min. Subsequently, the chamber was additionally exposed to UV light for 30 min.

Table 2 presents the concentrations of the studied volatile substance vapors at 20 °C. The concentrations were calculated using the following Formula (3):

(3) $$C_{g}=\frac{PM}{RT}$$

where *M* is the molar mass of the VOC, *Cg* is the saturated vapor concentration of the volatile compound in g/m^3^, *P* is the vapor pressure of the volatile compound, *R* is the universal gas constant, and *T* is the temperature.

For converting from grams per cubic meter (g/m^3^) to parts per million (ppm) units of concentration, the following Formula (4) was used:

(4) $$C=\frac{22.45\times C_{g}}{M}$$

where *C* is the vapor concentration expressed in ppm, 22.45 is the molar volume of gas at 25 °C, and *M* is the molar mass of the VOC.

### 2.6. Methodology for Calculating Sensor Response

The calculation of the sensor response of the films was carried out according to the methodology described in detail in the works [32]. The sensor response was calculated using the Formula (5):

(5) $$\alpha =\frac{\Delta S_{12}}{L},$$

where α is the normalized value of the sensor response, Δ*S*_12_ are the losses due to the interaction of the sensor film with the studied substance, calculated as the difference between the signal amplitude with and without the sensor layer on the delay line, and *L* is the length of the area covered by the sensor film.

The calculation of the phase response (*R*) was performed using the expression (6):

(6) $$R=\frac{\Delta \varphi \lambda }{360^{{}^{\circ}}L}\times 10^{6}$$

where *L* is the distance between the transducers, equal to 9 mm; *λ* is the wavelength of the transducers, equal to 34 μm, and Δ*φ* is the phase change resulting from the interaction of the detectable substance with the sensor film.

## 3. Results and Discussion

### 3.1. Surface Properties of Langmuir Monolayers of Tetra-Tert-Butylphthalocyaninate Zinc

**Figure 4:**
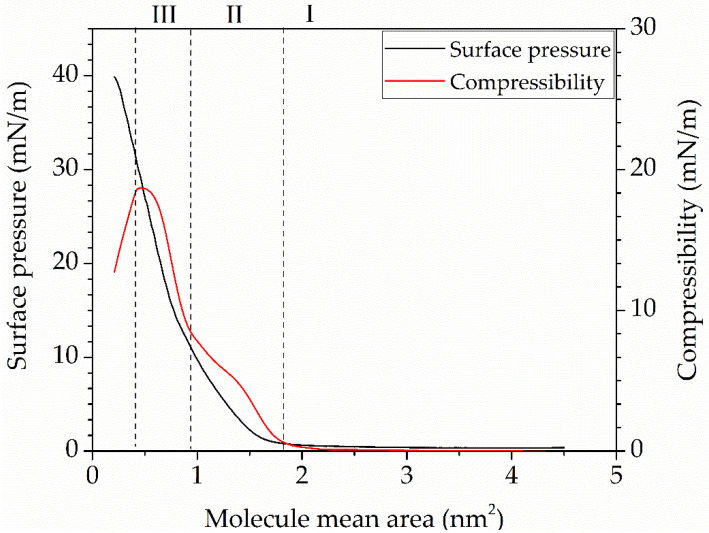
Compression isotherm (black curve) of tetra-tert-butylphthalocyaninate zinc monolayer and changing of the monolayer compressibility (red curve), where (I) is the gaseous phase, (II) is the tilted condensed phase and (III) is the untitled condensed phase.

The resulting compression isotherm of the tBuZnPc monolayer is presented in Figure 4. Tetra-tert-butylphthalocyaninate zinc is able to form a stable Langmuir monolayer on the water surface. On the compression isotherm, one can distinguish the gas (I), tilted condensed (II), and non-tilted condensed (III) phases of the monolayer. Figure 4 shows the graph of the monolayer compressibility change.

When the specific area per molecule (A) in the monolayer exceeds 1.1 nm^2^, the monolayer is in the gas phase. According to references [33,34], in the gas phase, the Langmuir monolayer has an island-like structure, where each island consists of planar aggregates of surfactant molecules. Compression of the monolayer changes the distance between the aggregates, leading to an increase in surface pressure when the area per molecule reaches values between 0.8 nm^2^ and 0.9 nm^2^. At this point, the monolayer transitions into the tilted condensed phase (II). The increase in surface pressure in phase (II) is attributed to intermolecular interactions between the tert-butyl groups of the phthalocyanine molecules. In the compressive modulus graph for region (II), an inflection point is observed at a compressive modulus of 7.5 mN/m and an area (A) of 0.55 nm^2^. The presence of this point may indicate a shift in the dominant type of intermolecular interaction from steric repulsion of the substituents to π-π interactions of the heterocyclic cores [35]. The subsequent phase transition to the non-tilted condensed phase (III) occurs at an area per molecule (A) of 0.7 nm^2^. In this phase, tBuZnPc molecules are oriented with their macrocycles at a 60° angle to the water surface, a configuration facilitated by the four substituent groups in the molecule. A further increase in monolayer compression is associated with structural disruption and eventual layer collapse [36].

The Langmuir monolayers were transferred onto solid substrates at a surface pressure of 19.5 mN/m, corresponding to the non-tilted condensed phase. In this state, the monolayer exhibited a structure with phthalocyanine molecules oriented at an angle of approximately 60° to the water surface. During the transfer process, a film with uniform morphology was expected to form, in which the π-conjugated system would be oriented at an angle of up to 30° to the substrate. This orientation of the phthalocyanines allows molecules of the analyte to access both the central atom of the phthalocyanine and its substituents, which should enhance the film’s sensitivity to target chemical vapor molecules.

### 3.2. Study of the Structutal Features of the Langmuir-Blodgett Films of Tetra-Tert-Butylphthalocyaninate Zinc

**Figure 5:**
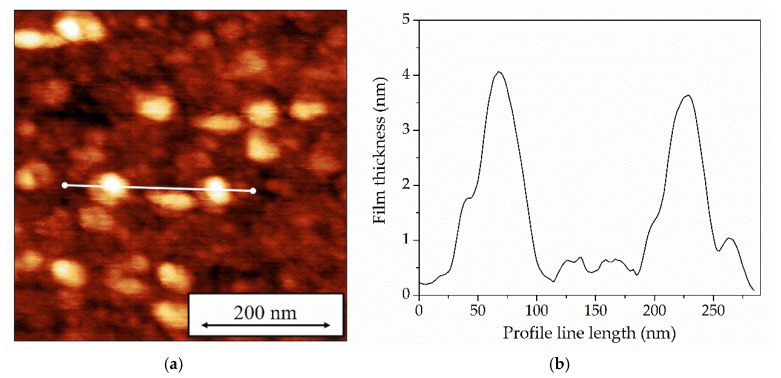
AFM image of the surface (**a**) of a Langmuir-Blodgett monolayer ZnPc film transferred onto a LiNbO_3_ plate at a surface pressure of 25 mN/m, and the surface profile line (**b**).

**Figure 6:**
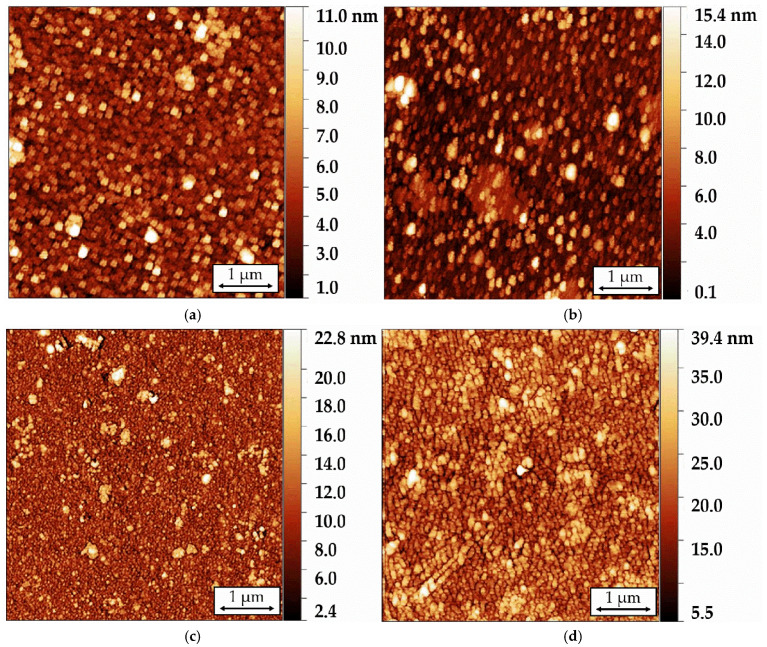
Typical images of the surface morphology of 5-layer (**a**), 11-layer (**b**), 21-layer (**c**), and 41-layer (**d**) sensor films of tetra-tert-butylphthalocyaninate zinc.

The study yielded surface morphology images of the films, as shown in Figure 5 and Figure 6. Figure 5 shows a typical image of a Langmuir-Blodgett monolayer film of tBuZnPc obtained by atomic force microscopy (AFM) in the hybrid mode, along with a surface profile line.

The figure reveals that the tBuZnPc film exhibits an inhomogeneous morphology with a surface roughness of approximately 1 nm. Columnar structures with heights of up to 4 nm are visible on the film surface. These structures likely formed during the monolayer transfer using the Langmuir-Blodgett method. At a surface pressure of 19.5 mN/m, the tBuZnPc monolayer possesses a stacked columnar structure of phthalocyanine molecules with an edge-on orientation. This finding is consistent with previous reports [37,38], where the authors investigated the orientation of phthalocyanine molecules with various substituents in Langmuir-Blodgett films formed at different surface pressures. The transfer of such a monolayer onto a solid substrate leads to the disruption of the stacked structures and the formation of a film in which the phthalocyanine macrocycles are oriented at small angles relative to the substrate. During the monolayer drying process, self-assembly of the phthalocyanine molecules occurs, resulting in the formation of aggregates of various sizes. Similar conclusions were reported by the authors in references [32,39] for floating Langmuir monolayers of tBuZnPc. To confirm the edge-on orientation, X-ray spectroscopy was employed.

The surface morphology of Langmuir-Blodgett sensor films comprising 5, 11, 21, and 41 tBuZnPc monolayers deposited on LiNbO_3_ piezoelectric plates was studied using atomic force microscopy (AFM). Figure 6 presents typical surface morphology images of the investigated films, acquired in the AFM hybrid mode. The final values of *P* and *Sa* listed in Table 3 were calculated as the average of the respective parameter from a series of measurements taken at five different points on the film.

The fabricated 5-layer film exhibits homogeneous surface morphology with an *S*a value of 1.3 nm and a surface area (*P*) of approximately 25.07 μm^2^. Increasing the number of layers to 11 raises *S*a to 1.4 nm and *P* to 25.15 μm^2^, with height variations on the film surface ranging from 10 to 12 nm. Further increasing the layer count to 41 results in *S*a and *p* values of 3.3 nm and 25.35 μm^2^, respectively. The simultaneous increase in both *S*a and *P* indicates the development of a more textured surface morphology. This growth in film heterogeneity with thickness may be attributed to defects introduced during the monolayer transfer process and the formation of phthalocyanine molecular aggregates.

The aggregation of phthalocyanine molecules was described in references [39,40], where thin films of various phthalocyanines were formed on muscovite mica surfaces. These studies noted a tendency for the formation of aggregates with heights comparable to the planar dimensions of phthalocyanine molecules. A similar aggregation effect was observed in the present work during the formation of multilayer films.

**Figure 7:**
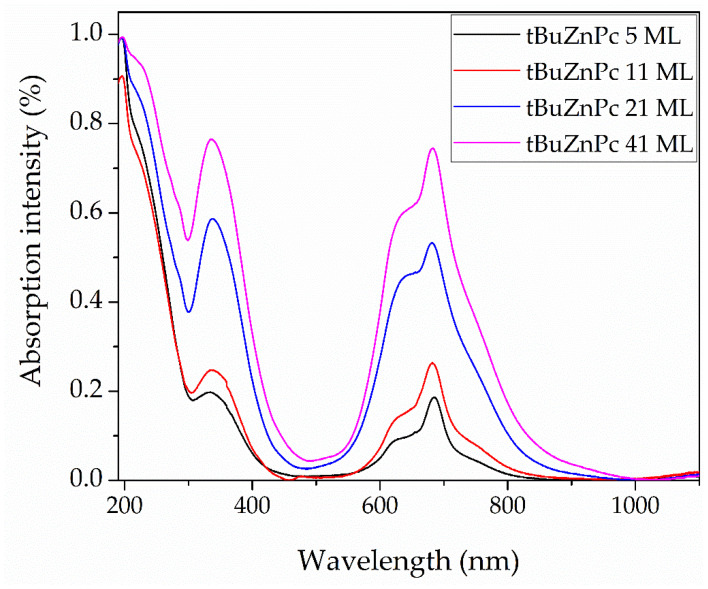
Absorption spectrum of tetra-tert-butylphthalocyaninate zinc films containing 5, 11, 21, and 41 Langmuir monolayers.

The observed morphological changes in the film surfaces also affect their optical properties. Analysis of Figure 6 and the data in Table 3 indicates that increasing the number of layers in the film increases its degree of heterogeneity. However, the AFM data do not provide clear information about changes in the orientation of the phthalocyanine molecules within the film. To investigate the effect of the number of layers on molecular orientation, the films were studied using UV-Vis transmission spectroscopy. Figure 7 shows the optical absorption spectra of tBuZnPc films with different numbers of monolayers. The spectra exhibit peaks corresponding to the Q and B absorption bands characteristic of phthalocyanine molecules. The positions and intensities of these absorption bands for each film are provided in Table 4.

As shown in Figure 7, the Q-band absorption is split into multiple components. This splitting pattern may indicate the formation of aggregates with non-parallel molecular alignment in the film [41]. Increasing the film thickness leads to an increase in the intensity of the Q-band in the absorption spectrum. Alongside this, the Q-band absorption widens.

The formation of a film with such morphology can be explained as follows. The first monolayer was transferred onto a substrate immersed perpendicularly to the water surface. When a surface pressure of 19.5 mN/m was achieved in the monolayer, the tBuZnPc molecules formed stacks oriented at an angle of approximately 60° relative to the water surface [35]. In this configuration, the angle between the substrate and the phthalocyanine molecules within the stacks is approximately 30°. During monolayer transfer, a water meniscus formed, altering the orientation angle of the stacks relative to the substrate surface. The absence of directional order in the phthalocyanine molecular stacks led to the formation of randomly shaped aggregates on the substrate surface during the transfer process. As the number of layers in the film increased, the shape and type of aggregates in the deposited layer became stabilized.

These conclusions are indirectly supported by analysis of the optical absorption spectra in Figure 7 and Table 4. For the five-layer film, the Q-band maximum is located at approximately 686 nm. With increasing film thickness, the Q-band maximum shifts and stabilizes at 682 nm for the eleven-monolayer film. This behavior of the Q-band absorption maximum suggests that further increases in film thickness do not substantially affect the molecular aggregation state within the film.

### 3.3. Sensor Properties of Tetra-Tert-Butylphthalocyaninate Zinc Films

**Figure 8:**
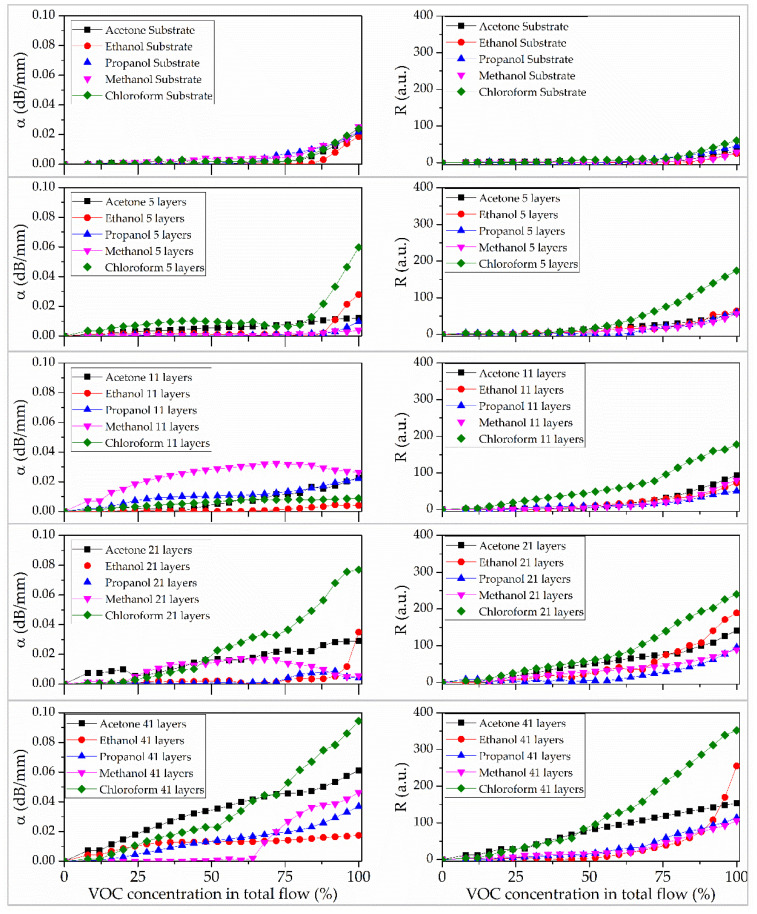
Concentration dependencies of the amplitude response (**left row**) and phase sensitivity (**right row**) of sensor coatings when interacting with vapors of studied liquids: without a sensor film (**first line**) and with sensor films containing five (**second line**), eleven (**third line**), twenty-one (**fourth line**), and forty-one (**fifth line**) ZnPc monolayers.

Figure 8 presents the sensor responses (R) and sensitivity (α) of the acoustic delay line both with and without films of different thicknesses upon exposure to volatile organic compound vapors. The acoustic delay line without a deposited sensor film exhibits a weak response, even at high concentrations of test vapors in the carrier gas. The maximum values of R and α are 25 a.u. and 0.02 dB/mm, respectively, observed at 100% saturation with test substance vapor. The detection threshold is approximately 75% of the saturated vapor concentration for all tested compounds.

The presence of sensor films on the acoustic delay line surface results in a selective response to chloroform vapor. Increasing the number of layers to 41 reduces the sensor’s detection threshold from approximately 312.5 ppm (80% of sample in total flow) to 40 ppm (10% of sample in total flow). Notably, the 41-layer film exhibits sensitivities of 150 a.u. to acetone vapor and 250 a.u. to ethanol vapor. The detection thresholds for ethanol and acetone were 20 ppm (50% of sample in total flow) and 13 ppm (6% of sample in total flow), respectively. For the 41-monolayer tBuZnPc film, the sensor response and recovery times were 2 s and 70 s, respectively.

**Figure 9:**
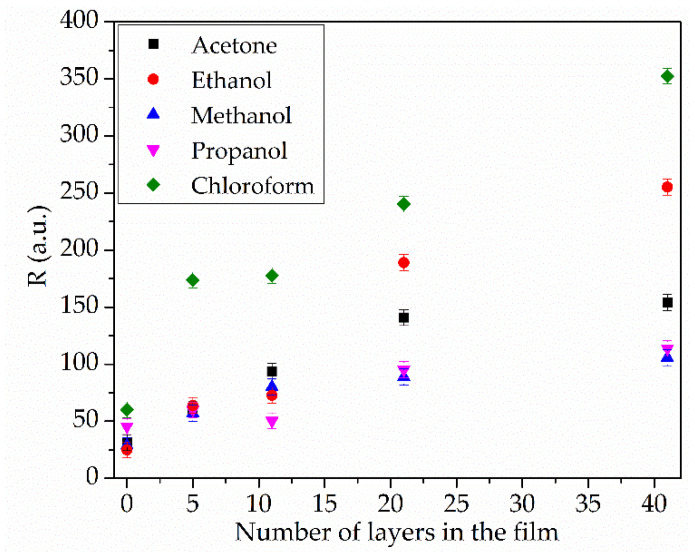
Dependence of the sensor response of the film on the number of layers at the maximum concentration of the analyzed gas.

Furthermore, an increase in film thickness alters its selectivity toward chloroform vapor. Figure 9 shows the dependence of the sensor response (R) on the number of film layers at the maximum detected gas concentration. As can be seen from the figure, the sensor response to the studied volatile substance vapors increases with coating thickness. In the absence of a sensor film, differences in the R values for the studied substances may be attributed to the varying thermodynamic effects these substances exert during their adsorption and evaporation on the plate surface [41].

The increase in the response (R) in the presence of a film is due to the interaction between the vapor of the studied substances and the developed surface of the sensor layer. An increase in the number of layers results in the most significant rise in the R value for chloroform vapor. The selectivity toward chloroform relative to other compounds is an important parameter. The coating comprising 5 tBuZnPc monolayers exhibits the highest selectivity factor of 2.9. An increase in response to ethanol, methanol, isopropanol, and acetone begins at a film thickness of 21 monolayers. This may indicate the onset of defect formation in the film; further growth of these defects enhances non-specific adsorption and the response to these solvents. Increasing the number of layers to 41 leads to a rise in R for all studied substances and a decrease in the selectivity index to 1.4.

The enhanced sensitivity of the fabricated coatings to chloroform vapor can be explained by several factors. Chloroform molecules exhibit high chemical affinity for tert-butyl substituent groups, enabling effective dissolution of tBuZnPc molecules in organic solvents like chloroform. Furthermore, the bulky tert-butyl groups can form hydrophobic cavities that preferentially capture nonpolar molecules [27,42,43]. It has been previously demonstrated that specific solvation of substituted phthalocyanine complexes can significantly alter their properties [44,45].

**Figure 10:**
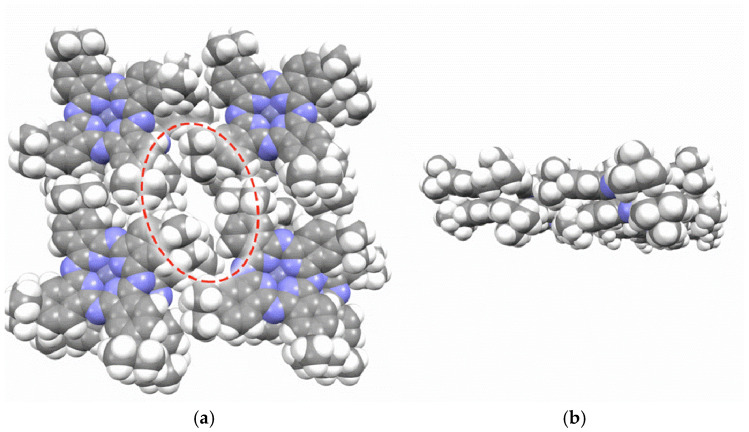
Schematic representation of the formation of hydrophobic cavities between tBuZnPc molecules in a Langmuir monolayer, (**a**) is a top-view, (**b**) is a bottom-view. The hydrophobic cavity is shown as the area outlined by dashed lines.

During compression of the tBuZnPc monolayer on the water surface, the molecules can reorganize from a flat to a more tilted configuration. Such reorganization may involve relative rotation of adjacent macrocycles and the formation of local nonpolar regions between the tert-butyl groups [29,46]. In these regions, the close packing of tert-butyl groups can lead to the formation of hydrophobic cavities. A schematic illustration of the formed hydrophobic cavities is presented in Figure 10.

To model the cavities formed by the tert-butyl groups in films of substituted zinc phthalocyanine, four molecules were arranged in a plane at the vertices of a square. A pair of these planes was then stacked to form a 2 × 4 fragment of a multilayer structure. This architecture is consistent with the Langmuir-Blodgett approach used to fabricate the sensing multilayers. Geometry optimization of the resulting 2 × 4 fragment revealed that the tert-butyl groups do form cavities, while the phthalocyanine molecular planes overlap due to π-stacking interactions. Therefore, the space between the phthalocyanine planes is unlikely to be responsible for solvent molecule binding. The geometry optimization was performed using the semiempirical tight-binding model GFN2-XTB in the ORCA 6.1.0 software package. The hydrophobic cavities formed between the tert-butyl groups represent nonpolar regions where nonpolar molecules can be trapped via van der Waals interactions. A similar mechanism has been described in detail in studies investigating the hydrophobic capture process [47,48,49].

**Figure 11:**
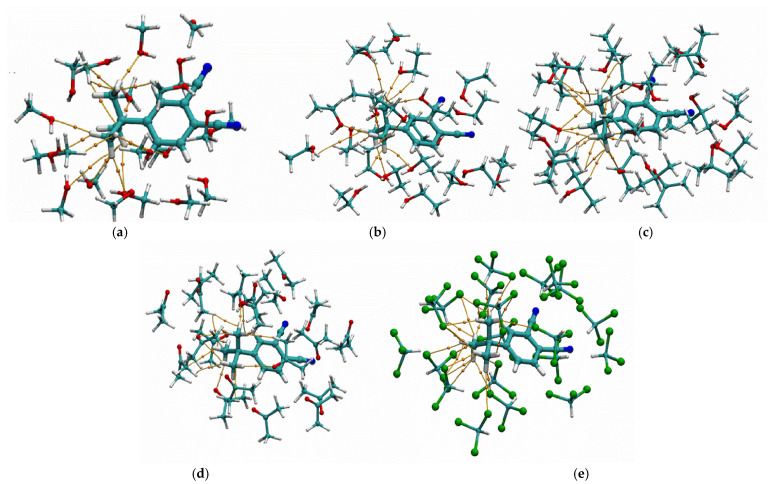
Visualization of interactions between the model molecule of tert-butylphthalonitrile and methanol (**a**), ethanol (**b**), isopropanol (**c**), acetone (**d**), and chloroform (**e**).

To evaluate the efficiency of the interaction between tert-butyl groups and target analyte molecules, the solvation process in various solvents—methanol, ethanol, isopropanol, acetone, and chloroform—was modeled. Since the interaction of polar molecules with the central atom of phthalocyanine has been extensively described in the literature [50], the model compound 4-tert-butylphthalonitrile, a phthalocyanine precursor, was used to simplify the calculations. For this purpose, the SOLVATOR algorithm implemented in ORCA software version 6.1.0 was employed [51]. A cluster consisting of one 4-tert-butylphthalonitrile molecule surrounded by 20 solvent molecules was generated using this algorithm. The geometry of the resulting solvation cluster was optimized using the semiempirical GFN2-xTB method. Subsequently, a single-point energy calculation was performed using the r2SCAN-3c composite density functional theory (DFT) method [52]. Wave function analysis was carried out with Multiwfn software version 3.8 to visualize non-covalent interactions between the tert-butyl groups and solvent molecules within the Quantum Theory of Atoms in Molecules (QTAIM) framework [53]. This enabled visualization of the paths connecting interacting atoms, and the potential energy *V*(*r*) at the corresponding bond critical points allowed estimation of the binding energy (*BE*) using the Espinosa–Molins–Lecomte correlation scheme [54]. Examples of the resulting visualizations are presented in Figure 11.

Figure 11 shows that for aliphatic alcohols and acetone, the tert-butyl groups are primarily solvated through van der Waals interactions (C–H, H–H), with a minor contribution from hydrogen bonding (O–H). The total interaction energies between 4-tert-butylphthalonitrile and the studied solvent molecules are given in Table 5.

As shown in Table 5, the total interaction energy for methanol, ethanol, isopropanol, and acetone ranges from –11.5 to –11.7 kcal/mol. In contrast, for chloroform, stronger H···Cl interactions increase the interaction energy to –18.7 kcal/mol.

**Figure 12:**
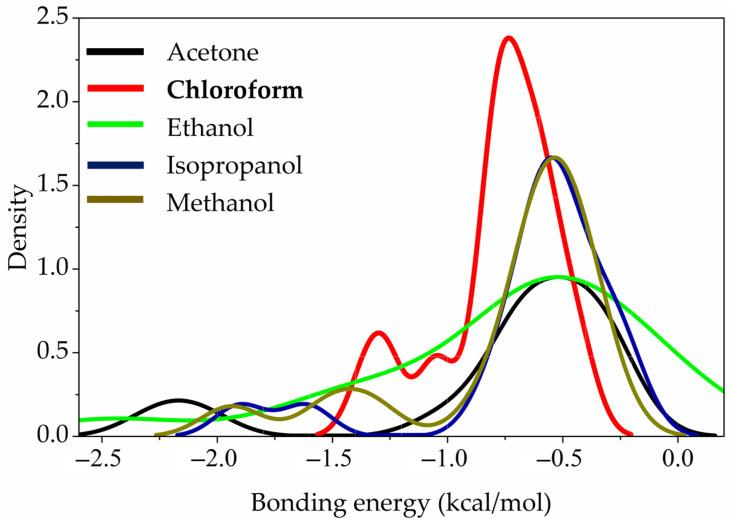
Distributions of bonding energies between vapor of chemistry molecules and tert-Butyl groups.

This feature can be clearly seen from the distributions of bonding energies that illustrated in Figure 12 in the studied systems. The global maximum of bonding energy at bond critical points in the case of chloroform is shifted to larger energies compared to the alcohols and acetone, while in the former case the stronger interactions have smaller contributions to the overall tert-butyl groups and solvent interaction energies.

An increase in the number of layers in the film alters the degree of ordering of the tBuZnPc molecules. This change leads to greater film roughness (*Sa*). Concurrently, microdefects and cracks form within the film. The presence of these structural irregularities increases the surface area (*P*) and creates additional sites for nonspecific adsorption of the studied compound molecules. Consequently, the response (*R*) to acetone, isopropanol, ethanol, and methanol vapors increases. In summary, the tert-butyl groups play a key role in the packing, orientation, and free volume of tBuZnPc Langmuir-Blodgett films, and therefore, in their optical and sensory properties.

**Figure 13:**
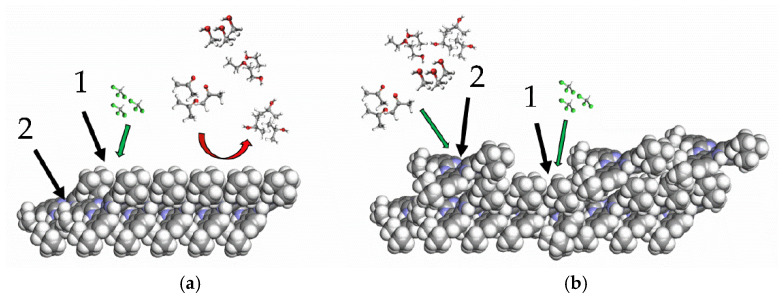
Schematic representation of response mechanism for a film thickness less than 11 layers (**a**) and more than 11 layers (**b**), where 1 is a hydrophobic cavities, 2 is a central atom of zinc in the phthalocyanine molecule.

The schematic mechanism of film sensitivity is shown in Figure 13.

For film thicknesses corresponding to approximately 5 and 11 monolayers of tBuZnPc, the dominant interaction mechanism between the analyte molecules and the film involves the van der Waals capture of nonpolar chloroform molecules within hydrophobic cavities. An increase in film thickness leads to greater structural defect density, which in turn enhances the accessibility of the central zinc atom in the macrocycle. This improved accessibility strengthens the interaction with more polar molecules, such as acetone and monohydric alcohols. Simultaneously, hydrophobic pockets continue to form within the film, and an increase in their number promotes further adsorption of chloroform molecules [17]. As a result, an overall increase in the sensor response of the film is observed.

## 4. Conclusions

Multilayer Langmuir-Blodgett films based on tetra-tert-butylphthalocyanine zinc were successfully fabricated on LiNbO_3_ acoustic delay lines and characterized. The films demonstrate selective sensitivity to chloroform vapor, showing a significantly stronger response compared to ethanol, acetone, isopropanol, and methanol. Films comprising 5 tBuZnPc monolayers exhibited the highest selectivity, which is attributed to specific interactions between chloroform molecules and the hydrophobic cavities formed by the tert-butyl groups of tBuZnPc. Increasing the film thickness to 41 layers enhanced the response to chloroform up to 370 a.u., but reduced selectivity due to increased nonspecific adsorption. The detection threshold for chloroform improved from 321 ppm to 39 ppm with increasing film thickness. Furthermore, thicker films developed a measurable response to polar compounds, specifically 250 a.u. for ethanol and 150 a.u. for acetone. These results demonstrate the potential for developing portable chloroform sensors for environmental monitoring and industrial safety applications. Langmuir-Blodgett films of tetra-tert-butylphthalocyanine zinc were used for the first time to achieve selective detection of chloroform vapor using acousto-electronic technology. The fabricated films are of interest for functionalizing the surfaces of multiparameter acoustic sensors. Such devices could form the basis of analytical systems for determining the composition of multicomponent liquids [55,56]. Their operation relies on analyzing histograms of acoustic response (HAR), which reflect changes in the amplitude of different types of acoustic waves upon interaction with the target liquid. Modifying the sensor surface with functional films enhances the discriminatory capability of HAR through selective interactions between target analyte molecules and the sensitive layer. Changes in film properties can uniquely affect acoustic waves depending on their propagation direction. Combining this approach with machine learning techniques creates opportunities for developing new-generation sensory systems with enhanced selectivity toward target molecules.

## Figures and Tables

**Table 1:** Comparison of the chloroform sensors.

Proposed Sensor	Sensitivity/Substance
Electrochemical sensor based on TiO_2_ nanotubes [2]	From 1000 ppm to 2000 ppm/Vapors
Electrochemical sensor based on ZnO nanorods covered by Fe_2_O_3_ colloidal suspension [3]	From 3.5 nM to 35 mM (4 × 10^−4^ to 4200 ppm)/Liquid
Electrochemical sensor based on Thin film of poly(3-decylthiophene-2,5-diyl) [4]	Depends on the concentration of active substance in the film./Vapors
Optical sensor based on thin film of ethylcellulose with incapsulated 2,2’-dipyridyl and tetrabutylammonium hydroxide [5]	Up to 0.830 ppm in non-aqueous solutions Up to 500 ppm in aqueous solutions/Liquids
Optical sensor based on thin film of 1,2,4,5-tetrapheny l-1,4-dihydropyrololo [3,2-6] pyrrole [6]	Up to 100 ppm/Vapors
Acoustic sensor based on LB films of fatty acids [7,8]	From 1.11 Hz/ppm to 7.72 Hz/ppm/Vapors
Acousto optic sensor based on chamber with laser and microphone system [19]	From 0.28 ppm/Vapors
Acoustooptic sensor based on porous gold film with immobilized enzyme molecules [20]	In the range of 9–12 Hz/ppm/Vapors

**Table 2:** Concentration of vapors of studied volatile substances at 20 °C.

	%	Isopropanol (C_3_H_8_O), ppm	Ethanol (C_2_H_5_OH), ppm	Methanol (CH_3_OH), ppm	Chloroform (CHCl_3_), ppm	Acetone (C_3_H_6_O), ppm
**Sample content in the flow, %**	2	1.2	0.7	0.8	7.8	4.4
	4	2.4	1.5	1.5	15.6	8.8
	6	3.6	2.3	2.3	23.4	13.1
	8	4.8	3.2	3.1	31.3	17.5
	10	6.1	3.9	3.8	39.1	21.9
	12	7.2	4.8	4.6	46.9	26.3
	14	8.5	5.6	5.3	54.7	30.7
	16	9.7	6.4	6.1	62.5	35.1
	18	10.9	7.1	6.9	70.3	39.4
	20	12.1	7.9	7.6	78.1	43.8
	22	13.3	8.7	8.4	85.9	48.2
	25	15.1	9.9	9.6	97.7	54.8
	28	16.9	11.1	10.7	109.4	61.4
	30	18.1	11.9	11.5	117.2	65.7
	35	21.1	13.9	13.4	136.7	76.7
	40	24.2	15.9	15.3	156.3	87.6
	45	27.2	17.9	17.2	175.8	98.6
	50	30.2	19.9	19.1	195.3	109.6
	60	36.2	23.8	22.9	234.4	131.5
	70	42.3	27.8	26.8	273.6	153.4
	80	48.3	31.8	30.6	312.5	175.3
	90	54.4	35.7	34.4	351.6	197.2
	100	60.4	39.7	38.2	379.1	213.9

**Table 3:** Average roughness (*Sa*) and average surface area (*P*) of the studied sensor films.

Number of Layers in the Film	*Sa*, nm	P, μm^2^
5	1.3 ± 0.2	25.07 ± 0.02
11	1.4 ± 0.3	25.15 ± 0.03
21	1.5 ± 0.4	25.18 ± 0.05
41	3.3 ± 0.3	25.35 ± 0.07

**Table 4:** Position and intensity of absorption for Q and B bands in tetra-tert-butylphthalocyaninate zinc films of different thicknesses.

Number of Layers	B-Band		Q-Band	
	Peak Position, nm	Transmission, %	Peak Position, nm	Transmission, %
5 layers	332	19	684	18
11 layers	336	25	682	26
21 layers	337	59	682	53
41 layers	338	76	680	74

**Table 5:** Total interaction energy between tert-butylphthalonitrile and methanol (MeOH), isopropanol (iPrOH), ethanol (EtOH), acetone (Acetone), and chloroform (CHCl_3_).

Solvent	Energy of Interaction, kcal/mol
MeOH	−11.8
iPrOH	−11.5
EtOH	−11.7
Acetone	−11.7
CHCl_3_	−18.7

## Data Availability

The original contributions presented in this study are included in the article. Further inquiries can be directed to the corresponding author.
